# Efficacy of Cefiderocol in Experimental Stenotrophomonas maltophilia Pneumonia in Persistently Neutropenic Rabbits

**DOI:** 10.1128/aac.00618-22

**Published:** 2022-09-26

**Authors:** Vidmantas Petraitis, Ruta Petraitiene, Povilas Kavaliauskas, Ethan Naing, Andrew Garcia, Benjamin N. Georgiades, Roger Echols, Robert A. Bonomo, Yoshinori Yamano, Michael J. Satlin, Thomas J. Walsh

**Affiliations:** a Transplantation-Oncology Infectious Diseases, Department of Medicine, Weill Cornell Medicine of Cornell University, New York, New York, USA; b Shionogi, Inc., Florham Park, New Jersey, USA; c Infectious Disease Drug Development Consulting, LLC, Easton, Connecticut, USA; d Medical Service, Louis Stokes Cleveland Department of Veterans Affairs Medical Center, and Departments of Medicine, Pharmacology, Molecular Biology, and Microbiology, Case Western Reserve University and Research Service, CWRU-VA Center for Antimicrobial Resistance and Epidemiology (CARES), Cleveland, Ohio, USA; e Pharmaceutical Research Division, Shionogi & Co., Ltd., Osaka, Japan; f Department of Pediatrics, Weill Cornell Medicine of Cornell University, New York, New York, USA; g Department of Microbiology and Immunology, Weill Cornell Medicine of Cornell University, New York, New York, USA

**Keywords:** cefiderocol, *Stenotrophomonas maltophilia*, pneumonia, trimethoprim-sulfamethoxazole, neutropenia

## Abstract

Stenotrophomonas maltophilia is an important cause of pneumonia in immunocompromised patients. Cefiderocol is a parenteral siderophore cephalosporin with potent *in vitro* activity against S. maltophilia. We evaluated the efficacy of cefiderocol in a neutropenic rabbit model of S. maltophilia pneumonia in comparison to trimethoprim-sulfamethoxazole (TMP-SMX). The cefiderocol area under the plasma drug concentration-time curve extrapolated to 8 h (AUC_0–8_) was lower (423.0 ± 40.9 μg·h/mL versus 713.6 ± 40.1 μg·h/mL) and clearance higher (252.77 ± 38.9 mL/h/kg versus 142.6 ± 32.9 mL/h/kg) in infected versus noninfected rabbits. We studied a clinical bloodstream S. maltophilia isolate with an MIC of 0.03 μg/mL of cefiderocol. Time spent above the MIC of cefiderocol for the majority of S. maltophilia isolates in rabbits recapitulated the plasma concentration-time profile observed in adult humans at the licensed dose of 2 g given intravenously (i.v.). Experimental groups consisted of 120 mg/kg cefiderocol i.v. every 8 hours (q8h); TMP-SMX, 5 mg/kg i.v. Q12h, and untreated controls (UCs). Treatment was administered for 10 days. Survival in cefiderocol-treated rabbits (87%) was greater than that in TMP-SMX-treated (25%; *P < *0.05) and UC (0%; *P < *0.05) groups. There was no residual bacterial burden in lung tissue or bronchoalveolar lavage (BAL) fluid in the cefiderocol group. Residual bacterial burden was present in lung tissue and BAL fluid in the TMP-SMX group but was decreased in comparison to UCs (*P < *0.001). Lung weights (markers of pulmonary injury) were decreased in cefiderocol-treated versus TMP-SMX (*P* < 0.001) and UC (*P < *0.001) groups. Cefiderocol is highly active in treatment of experimental S. maltophilia pneumonia, laying the foundation for future clinical investigations against this lethal infection in immunocompromised patients.

## INTRODUCTION

Stenotrophomonas maltophilia causes potentially lethal pneumonia, bacteremia, ecthyma gangrenosum, and sepsis in immunocompromised patients (1–4). The clinical manifestations of infections due to S. maltophilia in this population are similar to those caused by Pseudomonas aeruginosa. Mortality from S. maltophilia pneumonia remains tragically high, particularly in severely immunocompromised patients, such as transplant recipients and those with hematological malignancies.

The treatment of invasive S. maltophilia infections is challenging because of the limited antimicrobial therapies that have activity against this organism. The expression of two genes, *L1* and *L2*, that encode a metallo-β-lactamase and a serine-β-lactamase, respectively, inactivate penicillins, cephalosporins, and carbapenems (5). A recent study indicated that S. maltophilia was the most common carbapenem-resistant Gram-negative pathogen causing bacteremia in the United States (6).

The current standard of care for the treatment of invasive infections caused by S. maltophilia is trimethoprim-sulfamethoxazole (TMP-SMX) (1, 2, 7). However, the clinical utility of TMP-SMX is limited by its toxicities, including rash, cutaneous photosensitivity, Stevens-Johnson syndrome, hyperkalemia, acute kidney injury, and myelosuppression (8). These toxicities may be particularly problematic in immunocompromised patients, such as transplant recipients who may have preexisting kidney disease and/or decreased blood cell counts. Fluoroquinolones and tetracycline derivatives, such as minocycline, also have *in vitro* activity and potential clinical utility in the treatment of S. maltophilia infections (9, 10). However, the use of these agents is limited by the emergence of resistance (11). S. maltophilia can accumulate multidrug efflux pumps that reduce the activity of fluoroquinolones and tetracyclines (12), and many strains have *Smqnr* genes that further decrease the activity of fluoroquinolones (13). Furthermore, current dosing regimens for these agents often do not achieve pharmacokinetic-pharmacodynamic parameters that correlate with efficacy, even when organisms test susceptible according to current breakpoints of the Clinical and Laboratory Standards Institute (14, 15). Last, the activity of TMP-SMX and fluoroquinolones may be further diminished in immunocompromised patients who often receive these antimicrobials as prophylaxis against *Enterobacterales* and Pneumocystis infections. Thus, new antimicrobial therapies are urgently needed for infections caused by S. maltophilia.

Cefiderocol is a new parenteral siderophore cephalosporin that is transported through the outer cell membrane by mimicking a natural siderophore that then inhibits Gram-negative cell wall biosynthesis (16, 17). Cefiderocol has potent *in vitro* activity against S. maltophilia (18–23) that is related to its efficient transfer across the outer cell membrane as well as its stability to serine and metallo-β-lactamases (24). However, little is known about its *in vivo* activity, dosage, or duration of treatment needed against S. maltophilia pneumonia in immunocompromised hosts (25, 26). We therefore studied the pharmacokinetics and efficacy of cefiderocol in the persistently neutropenic rabbit model of S. maltophilia pneumonia.

## RESULTS

### *In vitro* antimicrobial susceptibility.

The MICs of cefiderocol (Shionogi & Co., Ltd., Osaka, Japan) against 12 clinical isolates of S. maltophilia ranged from ≤0.03 to >32 μg/mL, with a median MIC of 0.06 μg/mL. Ten of the tested isolates had cefiderocol MIC values ≤0.25 μg/mL. For trimethoprim-sulfamethoxazole (TMP-SMX; Bactrim; Teva Pharmaceuticals, North Wales, PA, USA), the MICs against S. maltophilia ranged from 0.25/4.75 μg/mL to 64/1,216 μg/mL, with a median value of 8/156 μg/mL. The isolate S. maltophilia 167-C3 (MIC of cefiderocol ≤ 0.03 μg/mL and MIC of TMP-SMX = 0.25/4.75), which was susceptible to cefiderocol and TMP-SMX, was selected for use in the rabbit model of experimental Stenotrophomonas maltophilia pneumonia in this study.

### Plasma pharmacokinetics of cefiderocol.

**FIG 1:**
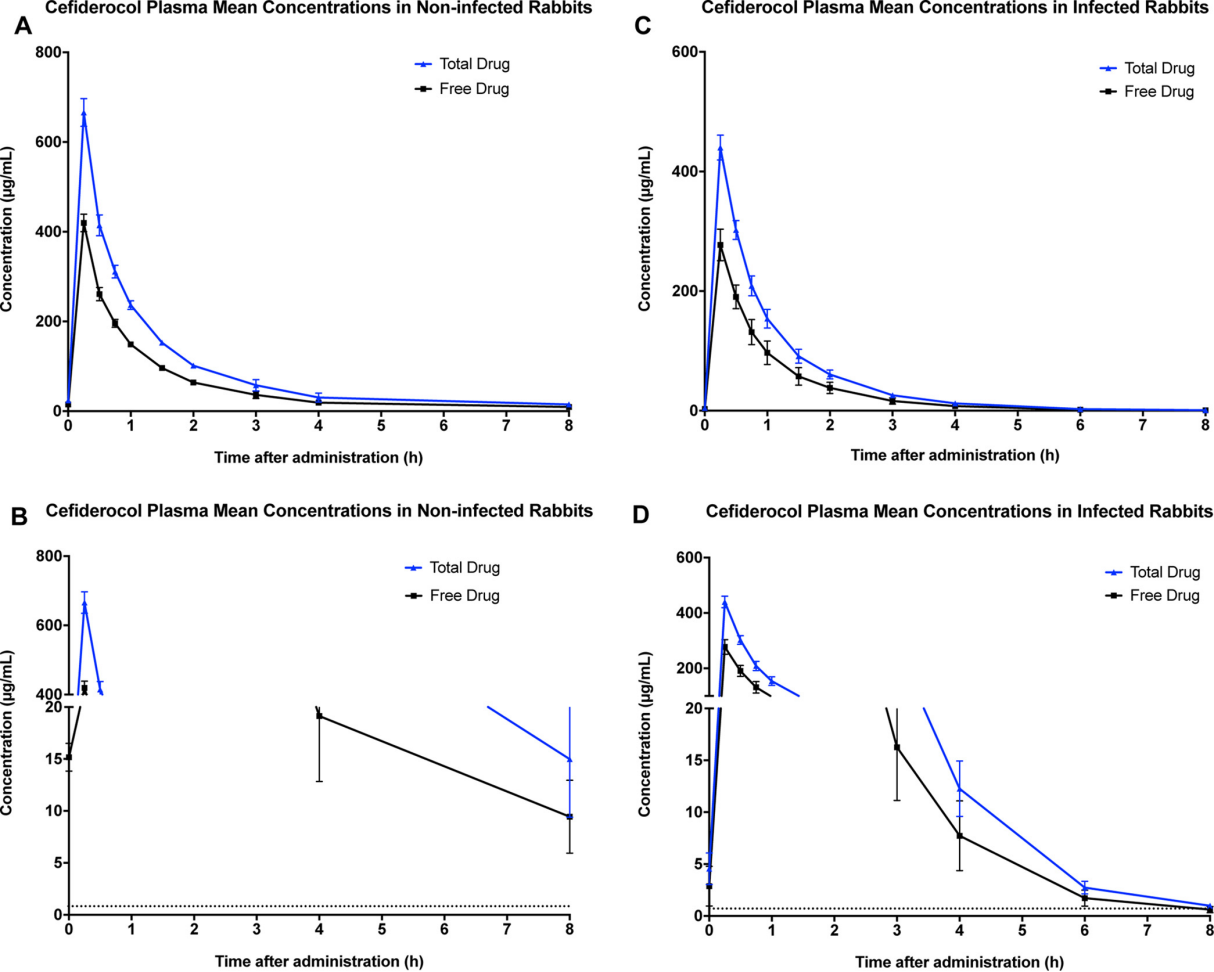
(A) Plasma concentration-time curves of total drug and free drug on day 3 of 120 mg/kg cefiderocol administered to noninfected NZW rabbits. (B) Plasma concentration-time curves of total drug and free drug on day 3 of 120 mg/kg cefiderocol administered to persistently neutropenic NZW rabbits with pneumonia from *Stenotrophomonas*. (C) Magnified lower portion of concentration-time curve demonstrating time above the MIC (0.03 μg/mL) of total drug and free drug on day 3 of 120 mg/kg cefiderocol administered to noninfected NZW rabbits. (D) Magnified lower portion of concentration-time curve demonstrating time above the MIC (0.03 μg/mL) of total drug and free drug on day 3 of 120 mg/kg cefiderocol administered to persistently neutropenic NZW rabbits with S. maltophilia pneumonia. All values are presented as means from samples of four rabbits each ± SEM.

The plasma concentration-time curves of cefiderocol over 8 h in noninfected rabbits and infected neutropenic rabbits are depicted in Fig. 1, and the noncompartmental pharmacokinetic parameters are presented in Table 1. The area under the plasma drug concentration-time curve extrapolated to 8 h (AUC_0–8_) was lower and the clearance was higher in infected rabbits than those parameters for noninfected rabbits. The plasma protein binding ratios determined by ultrafiltration were 38.5% and 36.8% at concentrations of 10 μg/mL and 100 μg/mL, respectively. Based on the cefiderocol MIC of 0.03 μg/mL for the S. maltophilia isolate used in the experimental model, the time above the MIC for free drug was 100% of the dosing interval.

**TABLE 1:** Pharmacokinetic parameters of cefiderocol after multiple intravenous administrations of 120 mg/kg q8h on day 3 in noninfected and infected with S. maltophilia neutropenic rabbits

Cefiderocol dose (mg/kg q8h)	Rabbit group	AUC_0–8_ (μg·h/mL)	*C*_max_ (μg/mL)	CL (mL/h/kg)	*V* (L/kg)
120	Noninfected	713.6 ± 40.1	666.0 ± 31.0	142.6 ± 32.9	218.7 ± 32.8
120	Infected	423.0 ± 40.9	440.01 ± 31.0	252.77 ± 38.9	310.64 ± 34.8

The mean AUC_0–8_ (standard error of the mean [SEM]) of 120 mg/kg cefiderocol administered intravenously (i.v.) over 10 min to infected rabbits in this study was 423.0 ± 40.9 μg·h/mL. This AUC_0–∞_ is comparable to the mean AUC_0–8_ (percent coefficient of variation [%CV] of the geometric mean) of 386 (17) μg·h/mL in humans when cefiderocol was dosed at the FDA-approved dosage of 2,000 mg as a 3-h infusion. The mean peak cefiderocol concentration (*C*_max_) (SEM) of 440 ± 31 μg/mL was higher in our rabbit model than that observed in humans (89 [20] μg·h/mL), reflecting the higher rate of infusion in the rabbit model.

### Outcome variables.

**FIG 2:**
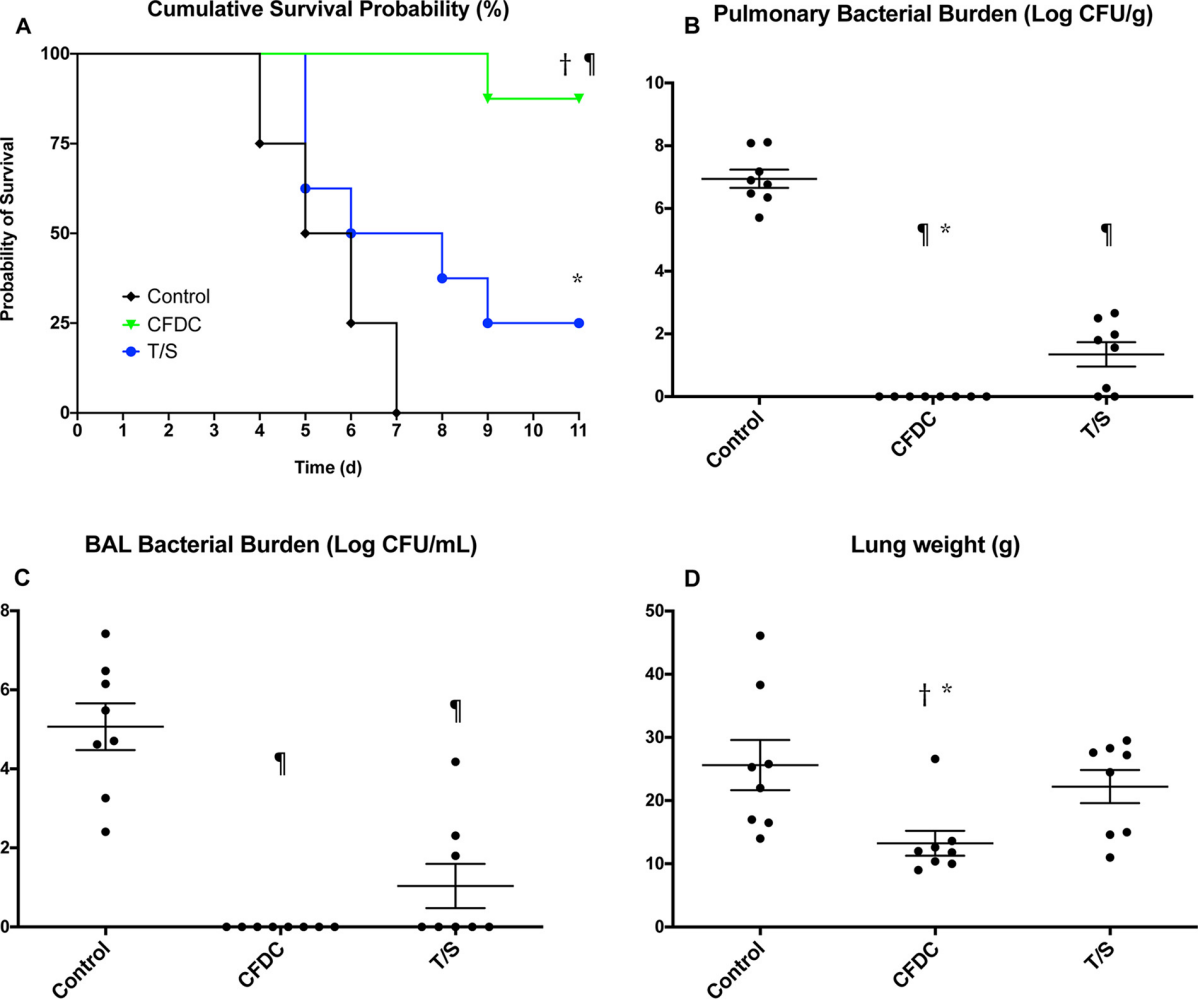
Response of S. maltophilia pneumonia in persistently neutropenic rabbits to treatment with cefiderocol (CFDC) (*n* = 8) and trimethoprim-sulfamethoxazole (TMP-SMX) (*n* = 8) measured by survival, pulmonary bacterial burden, BAL fluid bacterial burden, and lung weights in comparison to untreated controls (UCs) (*n* = 8). Values are given as means ± SEMs. Survival values are expressed as percentage of cumulative survival probability. Survival was plotted by Kaplan-Meier analysis. Comparisons of survival of treatment groups and UCs were analyzed by log-rank test. *P* values are indicated as follows: ¶, *P < *0.001, prolonged survival in cefiderocol-treated rabbits in comparison to that of UC; *, *P < *0.05, prolonged survival in trimethoprim-sulfamethoxazole-treated rabbits in comparison to that of control. For mean pulmonary tissue and BAL fluid residual bacterial burden and lung weights, ¶, *P < *0.001, decreased residual bacterial burden in CFDC- and TMP-SMX-treated rabbits versus that of control; *, *P* < 0.05, decreased pulmonary tissue residual bacterial burden of CFDC-treated rabbits versus that of TMP-SMX treated rabbits; †, *P* < 0.01, decreased lung weights in CFDC-treated rabbits versus those of control; *, *P* < 0.05, decreased lung weights of CFDC-treated versus those of TMP-SMX-treated rabbits.

**(i) Survival.** Survival to 11 days in rabbits treated with cefiderocol and TMP-SMX was significantly prolonged in comparison to that of untreated rabbit controls (UCs) (cefiderocol versus UCs, *P < *0.001; TMP-SMX versus UCs, *P < *0.05). In addition, significantly prolonged survival was achieved in cefiderocol-treated rabbits in comparison to those treated with TMP-SMX (*P < *0.05) (Fig. 2).

**(ii) Residual bacterial burden and lung weights.** The mean residual pulmonary bacterial burden in lung tissue and bronchoalveolar lavage (BAL) fluid was below the limit of detection in cefiderocol-treated rabbits (Fig. 2) compared to 6.13 ± 0.34 log CFU/g in lung tissue and 5.07 ± 0.59 log CFU/mL in BAL fluid in untreated controls (*P < *0.001 for comparisons with cefiderocol). The mean residual bacterial burden in TMP-SMX-treated rabbits was 1.28 ± 0.44 log CFU/g in lung tissue and 1.04 ± 0.56 log CFU/mL in BAL fluid, which was greater than the residual burden in cefiderocol-treated rabbits (*P < *0.05 for both comparisons) but less than the residual burden in untreated controls (*P < *0.05 for both comparisons).

There also was a significant reduction in mean lung weights, which are markers of organism-mediated pulmonary injury, of rabbits treated with cefiderocol in comparison to those of UCs (cefiderocol, 13.25 ± 1.98 g; UCs, 25.63 ± 3.98 g; *P < *0.01) (Fig. 2). In contrast, mean lung weights of TMP-SMX-treated rabbits (22.21 ± 2.62 g) were not significantly different from those of untreated controls.

**(iii) Detection of emergence of resistance.** Blood cultures were negative throughout the study. No S. maltophilia isolates were recovered in cefiderocol-treated rabbits to assess for the emergence of resistance in therapy. Organisms recovered from BAL fluid and lung tissue did not display emergence of resistance to either cefiderocol or to TMP-SMX.

**FIG 3:**
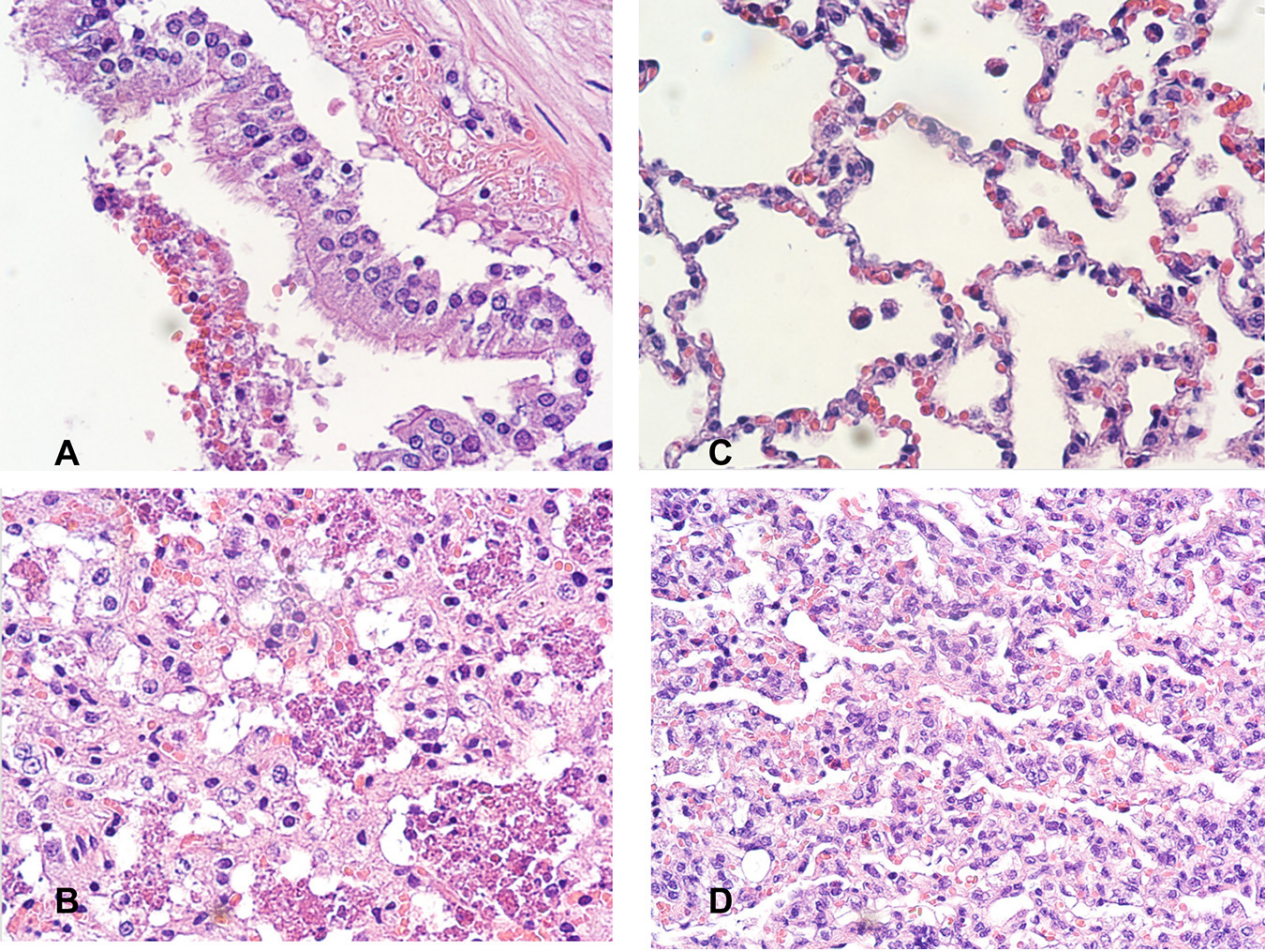
Histopathology of lung tissue of persistently neutropenic rabbit model of Stenotrophomonas maltophilia pneumonia. (A) Untreated control demonstrates disrupted tracheobronchial mucosa with epithelial necrosis and subepithelial inflammation in untreated controls (original magnification, ×400; H&E). (B) Extensive intra-alveolar infiltration with macrophages, mononuclear immune cells, proteinaceous exudates; and aggregates of intra-alveolar bacilli in untreated controls (original magnification, ×400; H&E). (C) In cefiderocol-treated rabbits, alveolar architecture is structurally intact; alveolar spaces contain minimal proteinaceous exudates and sparse macrophages, while macrophages and mononuclear cells are observed within the interstitium, and no bacteria are visible (original magnification, ×400; H&E). (D) In TMP-SMX-treated rabbits, lung tissue demonstrates preservation of the alveolar architecture and alveolar spaces with minimal proteinaceous exudates, sparse macrophages, and no histologically evident bacteria; however, there remained relatively large numbers of macrophages and mononuclear cells within the interstitium consistent with a residual inflammatory response (original magnification, ×400; H&E).

**(iv) Histopathology.** The histopathological features of bacterial S. maltophilia pneumonia were studied in the lungs of treatment groups and untreated controls. Figure 3A and B demonstrate disrupted tracheobronchial mucosa and subepithelial necrosis, as well as extensive alveolar infiltration with macrophages, mononuclear immune cells, proteinaceous exudates, and aggregates of intra-alveolar bacilli in untreated controls. By comparison, lung tissue from cefiderocol-treated rabbits revealed that the alveolar architecture was retained (Fig. 3C), alveolar spaces contain minimal proteinaceous exudates and sparse macrophages, macrophages and mononuclear cells were observed within the interstitium, and no bacteria were visible. Lung tissue from TMP-SMX-treated animals (Fig. 3D) also demonstrated preservation of the alveolar architecture and alveolar spaces with minimal proteinaceous exudates, sparse macrophages, and no histologically evident bacteria; however, there remained relatively large numbers of macrophages and mononuclear cells within the interstitium, consistent with a residual inflammatory response.

## DISCUSSION

Stenotrophomonas maltophilia causes life-threatening pneumonia, bloodstream infections, and sepsis in immunocompromised patients. This study demonstrated that cefiderocol achieved complete clearance of Stenotrophomonas maltophilia from lung tissue and bronchoalveolar lavage fluid in a persistently neutropenic rabbit model of Stenotrophomonas maltophilia. In contrast, TMP-SMX reduced residual pulmonary and BAL fluid bacterial burden compared to untreated controls but did not completely eliminate the organisms. In addition to microbial reduction, cefiderocol-treated animals demonstrated a marked reduction of lung weights as markers of organism-mediated pulmonary injury in comparison to those of TMP-SMX-treated animals and untreated controls. Coinciding with the significant reduction of lung weights, cefiderocol also significantly improved survival in comparison to that of untreated control animals and TMP-SMX-treated rabbits. By comparison, TMP-SMX-treated animals did not achieve a significant reduction in lung weights, which may have contributed to the lower survival rate than that of cefiderocol. These studies provide a foundation for further clinical investigation of cefiderocol in the primary treatment of serious and life-threatening S. maltophilia pneumonia, particularly in immunocompromised patients.

The cefiderocol dosage of 120 mg/kg administered every 8 h (q8h) of cefiderocol provided free drug time above the MIC that covered 100% of the dosing interval for the infecting pathogen with an MIC of 0.03 μg/mL. Even if one evaluated a strain with a cefiderocol MIC that was 1 μg/mL (a value higher than the MIC_90_ of 0.25 to 0.5 μg/mL identified in surveillance studies) (22, 27), the dosage of 120 mg/kg every 8 h would still provide nearly complete time above the MIC. The AUCs achieved in the rabbit model coincide with similar pharmacokinetic parameters to those in human studies (17), indicating that the licensed dose of cefiderocol of 2 g i.v. q8h in adults would also provide a similar time above the MIC in human adult patients. One should note, however, that these targets are for serum and not necessarily for pneumonia (28). Additional studies are warranted to address the pharmacokinetic/pharmacodynamic (PK/PD) targets in pneumonia.

The rabbit model Stenotrophomonas maltophilia in the persistently neutropenic rabbits simulates that of the human infection. Both cefiderocol and TMP-SMX produced significant clearance of organisms from both lung tissue and BAL fluid. However, the persistence of pulmonary injury, as measured by greater lung weights and histological evidence of macrophages and mononuclear cells, as well as the greater residual bacterial burden in the TMP-SMX-treated animals, may have contributed to the differences in survival. Ongoing sepsis and inflammation from residual organisms and the organism-mediated pulmonary injury may have led to the survival differences.

Stenotrophomonas maltophilia is well-known to develop resistance during the course of therapy for serious infections, including pneumonia. No organisms were recovered from lung tissue or BAL fluid from cefiderocol-treated rabbits. Organisms isolated from BAL fluid and lung tissue of TMP-SMX-treated rabbits did not demonstrate the emergence of resistance to TMP-SMX, suggesting that the emergence of resistance did not contribute to the comparatively worse outcome measures in the TMP-SMX group than the cefiderocol group.

There are several limitations to this study. First, the plasma pharmacokinetics of TMP-SMX were not determined in this model. As little is known about the pharmacokinetics of TMP-SMX in rabbits, we made the best estimate based upon limited literature and the experience of colleagues. Clearly, more work is required for the study of plasma pharmacokinetics of TMP-SMX in this model, and the differences in comparative outcome between cefiderocol and TMP-SMX should be regarded cautiously. Another limitation is the use of a single strain of S. maltophilia. Although we considered that the single isolate of S. maltophilia would be the most representative by MICs, other variables, including virulence factors, may also contribute to outcome in the model. Finally, the study of other isolates with higher MICs to cefiderocol and TMP-SMX would also help to define its role in more resistant organisms.

These data provide an important experimental rationale for the study of cefiderocol as therapy for immunocompromised patients with S. maltophilia. Very few patients in registrational randomized trials of cefiderocol were infected with S. maltophilia, precluding any conclusions of efficacy in these trials (29, 30). Furthermore, prospective observational studies to characterize the effectiveness of cefiderocol for treatment of serious Stenotrophomonas maltophilia infections are limited (31). The *in vivo* efficacy identified in this study provides evidence to support a clinical trial of cefiderocol for S. maltophilia infections in immunocompromised patients. Moreover, a randomized trial of cefiderocol versus TMP-SMX would further define the efficacy and safety of this novel cephalosporin in the primary treatment of serious Stenotrophomonas maltophilia infections.

## MATERIALS AND METHODS

### Study drugs.

Cefiderocol provided by Shionogi & Co., Ltd. (lot number 12M01) was compared to trimethoprim-sulfamethoxazole (Teva Pharmaceutical Industries Ltd.; lot number ST1324; catalog no. 106680), which is the current standard of care for the treatment of S. maltophilia pneumonia in neutropenic hosts.

### Bacterial isolates and *in vitro* antimicrobial susceptibility.

Clinical S. maltophilia isolates derived from patients with documented *Stenotrophomonas* bacteremia were used in the studies. Cefiderocol MIC values were determined for 12 bloodstream isolates of S. maltophilia by standard broth microdilution (BMD) methods using iron-depleted, cation-adjusted Mueller-Hinton broth (ID-CAMHB) (International Health Management Associates, Inc., Schaumburg, IL, USA) (32). BMD MIC values were also determined for TMP-SMX by using CAHMB. The quality control (QC) strain of Escherichia coli ATCC 25922 was tested each day of testing MICs. The range of studied concentrations for cefiderocol was 0.03 μg/mL to 32 μg/mL, and for TMP-SMX, it was 0.063/1.19 μg/mL to 64/1,216 μg/mL. MICs were calculated from four replicate wells. The S. maltophilia isolate 167-C3, which was susceptible to cefiderocol (MIC ≤ 0.03 μg/mL) and to TMP-SMX (MIC = 0.25/4.75 μg/mL), was selected as the candidate organism for *in vivo* studies.

Bloodstream isolates were selected for their unequivocal demonstration of virulence in patients. Isolate 167-C3 was selected among the 12 isolates for the lowest combined susceptibility profiles of cefiderocol and TMP-SMX.

### Animals.

Female New Zealand White (NZW) rabbits weighing 2.7 to 3.5 kg were used in all experiments. Care of rabbits in the laboratory animal facility was conducted according to guidelines of the Association for Assessment and Accreditation of Laboratory Animal Care and approved by the Weill Cornell Medicine Institutional Animal Care and Use Committee (IACUC). Rabbits were individually housed, and food and water were provided *ad libitum*. All rabbits had an indwelling intravenous catheter that was placed while they were under general anesthesia (33).

The rabbit model of S. maltophilia pneumonia affords distinctive advantages over previous animal model systems. As S. maltophilia pneumonia carries the highest mortality and morbidity in severely immunocompromised patients, assessment of new antimicrobial agents, such as cefiderocol, warrants study in animal model systems that reflect this profound level of immune impairment. The profoundly persistently neutropenic rabbit model of S. maltophilia recapitulates the histological and microbiological features observed in patients (34). Animals are persistently neutropenic (absolute neutrophil count [ANC] < 500 white blood cells [WBCs]/mm^3^) for 10 days. The model produces a multilobar bronchopneumonia that histologically consists of disrupted tracheobronchial mucosa and infiltration by activated intra-alveolar macrophages (Fig. 3). The indwelling intravenous catheter permits the atraumatic acquisition of diagnostic and pharmacokinetic samples, as well as parenteral administration of investigational antimicrobial agents, and compounds required to induce and support neutropenia. Further underscoring the value of the rabbit model in studying TMP-SMX in bacterial infections are key differences in thymidine concentrations among mammals (35). The median serum levels of thymidine in rabbits, dogs, and rhesus monkeys approximate those of humans, while median serum thymidine levels in rodents, including mice and rats, are approximately 4- to 6-fold higher.

### Pharmacokinetics.

**(i) Blood sampling, processing, and storage.** Cefiderocol was administered as a 10-min intravenous infusion at a dosage of 120 mg/kg every 8 h. Blood samples were obtained for pharmacokinetic analysis in noninfected rabbits (*n* = 4) on day 3 of antimicrobial therapy at the following time points: baseline, postinfusion (15 min, 30 min, 45 min, 1 h, 1.5 h, 2 h, 3 h, 4 h, and 8 h), and 15 min after the last dose on day 4. The purpose of studying pharmacokinetics in noninfected rabbits was to understand the impact of infection on the PK parameters of cefiderocol. Blood samples were obtained for pharmacokinetic analysis in rabbits infected with S. maltophilia (*n* = 4) on day 3 postinoculation and antimicrobial therapy at the following time points: baseline and postinfusion (15 min, 30 min, 45 min, 1 h, 1.5 h, 2 h, 3 h, 4 h, 6 h, and 8 h). Blood samples were obtained for pharmacokinetic analysis at the end of the study before euthanizing the infected rabbits 15 min after the last dose (day 4). Blood was collected via the established vascular access into 3-mL heparinized syringes, transferred into 15-mL polypropylene conical Falcon tubes (Becton, Dickinson Labware, Franklin Lakes, NJ), and separated via centrifugation at 400 × *g* for 10 min at 4°C. Plasma was stored at approximately −80°C in 2 mL Sarstedt microtubes until they were analyzed.

**(ii) Determination of cefiderocol concentrations.** The plasma concentrations of cefiderocol in noninfected rabbits were determined by Shionogi & Co., using validated liquid chromatography-tandem mass spectrometry (LC-MS/MS) (36). Cefiderocol plasma concentrations in infected rabbits were determined by Keystone Bioanalytical, Inc., (North Wales, PA) using LC-MS/MS. Briefly, the LC-MS/MS system consisted of an LC-20A high-performance liquid chromatography (HPLC) system (Shimadzu Corporation) in tandem with a Sciex API 5000 triple-quadrupole mass spectrometer in electrospray ionization mode (https://sciex.com/products/mass-spectrometers/triple-quad-systems/). Cefiderocol concentrations were determined by LC-MS/MS monitoring of product ion transitions of *m/z* 752.0 and *m/z* 285.0. The lower limit of quantification of cefiderocol was 0.1 μg/mL. Plasma protein binding was determined by ultrafiltration, as previously described (37).

**(iii) Pharmacokinetic analysis.** The pharmacokinetic profile for cefiderocol was computed from the concentration-time data using noncompartmental methods. The peak cefiderocol concentration (*C*_max_) was obtained directly from the observed data. The area under the plasma cefiderocol concentration-time curve from 0 to 8 h (AUC_0–8_) was calculated by use of the log-linear trapezoidal rule. Total body clearance (CL) was obtained from the equation dose/AUC_0–8_. The terminal elimination rate constant (*k*_el_) was obtained from a log-linear regression of the plasma concentration compared to time data in the terminal postdistribution phase. The volume of distribution (*V*) for cefiderocol was calculated as *V* = CL/*k*_el_.

### Inoculation.

Isolate 167-C3 that was susceptible to cefiderocol and TMP-SMX was used to establish S. maltophilia pneumonia. In PK studies with infected rabbits (*n* = 4), the concentration of bacteria was adjusted in order to give each rabbit a predetermined inoculum of 1.0 × 10^8^ CFU in a volume of 250 to 350 μL.

The concentration of inocula for the treatment experiments was adjusted to 1.0 × 10^10^ CFU of S. maltophilia for each rabbit in a volume of 250 to 350 μL. Inoculation with S. maltophilia was performed on day 2 of the experiment in order to establish colonization of the respiratory tract as the rabbit enters neutropenia. Each rabbit was anesthetized with 0.5 to 0.7 mL of a 2:1 mixture (vol/vol) of intravenous 100 mg/mL ketamine (Ketaset; Phoenix Scientific, Inc., St. Joseph, MO) and xylazine 20 mg/mL (Rompun; Bayer Corporation, Agriculture Division, Animal Health, Shawnee Mission, KS) for analgesia, amnesia, and skeletal muscle relaxation. Anesthetic dosage was adjusted according to body weight in order to achieve similar depths to general anesthesia. Once satisfactory anesthesia was obtained, a Flagg O straight-blade laryngoscope (Welch-Allyn Inc., Skaneateles Falls, NY) was inserted into the oral cavity until the vocal cords were clearly visible. The inoculum was then administered endotracheally with a tuberculin syringe attached to a 5.25-inch 16-gauge Teflon catheter (Becton, Dickinson Infusion Therapy Systems Inc., Sandy, UT). Concentrations of inocula were confirmed by serial dilution and culture on tryptic soy agar with 5% sheep blood (SBA) plates.

### Immunosuppression of rabbits and supportive care.

Cytarabine (Ara-C; distributed by Hospira, Inc., Lake Forest, IL, made in Japan) was initiated 1 day before the endotracheal inoculation of the animals. Total leukocyte counts and the percentages of granulocytes were monitored twice weekly with a Coulter counter (Coulter Corporation, Miami, FL) and by use of peripheral blood smears and differential counts, respectively. Profound and persistent neutropenia (<100/μL) was achieved by an initial course of 525 mg/m^2^ of Ara-C on days 1 through 5. Ara-C was administered at 484 mg/m^2^ on days 8 to 9 and day 13 of the experiment to maintain profound and persistent neutropenia. Cytarabine sterile solution for injection at 50 mg/mL was used in the studies.

Methylprednisolone (Solu-Medrol; distributed by Pharmacia & Upjohn Co., Division of Pfizer Inc.) was administered at 5 mg/kg of body weight on days 1 and 2 of the experiment to inhibit macrophage activity and to facilitate establishment of infection. Methylprednisolone sodium succinate for injection was distributed in vials of 2 mL containing 125 mg of methylprednisolone. The diluted solution was prepared by diluting 2 mL of concentrate in 18 mL of normal saline (1 mL = 6.25 mg of methylprednisolone). The diluted solution of methylprednisolone was stored at 4°C and discarded after 48 h.

Vancomycin (manufactured for Athenex, Schaumburg, IL) at 15 mg/kg was administered intravenously daily from day 4 of chemotherapy until study completion for prevention of opportunistic Gram-positive bacterial infections. Oral vancomycin was administered in drinking water at 50 mg/L in order to prevent the development of diarrhea caused by Clostridium spiroforme.

### Antimicrobial therapy.

Experimental groups for the efficacy study consisted of cefiderocol administered at 120 mg/kg intravenously q8h over 10 to 15 min (CFDC; *n* = 8), trimethoprim-sulfamethoxazole administered at 5 mg/kg intravenously Q12h over 10 to 15 min (TMP-SMX; *n* = 8), and untreated control rabbits (control; *n* = 8). Antimicrobial therapy was initiated 8 h after direct endotracheal inoculation and continued for 10 days.

The dosage of cefiderocol in rabbits was based upon human-equivalent dosages (HEDs) determined by allometric scaling based upon body surface area, as well as assessment of dosages used in other experimental and clinical studies. The dosage of cefiderocol was then further verified by determination of plasma concentration-time curves, protein binding by ultrafiltration, and assessment of time above the MIC through the dosing interval. The dosage of TMP-SMX is that used in humans for the treatment of serious Gram-negative infections. Higher dosages of TMP-SMX were not used empirically for concern of additive myelosuppression in profoundly neutropenic animals. Plasma concentrations were not determined for TMP-SMX.

### Outcome variables.

**(i) Survival.** Survival time in days postinoculation was recorded for each rabbit in each group. Following humane endpoints, rabbits were euthanized by intravenous administration of pentobarbital (65 mg of pentobarbital sodium/kg of body weight). Surviving rabbits were euthanatized by sodium pentobarbital anesthesia on day 10 postinoculation.

**(ii) Quantitative cultures of lung tissue.** Lung tissues from each rabbit were sampled and cultured by standard excision of tissue from each lobe. Each tissue sample was weighed, placed in a sterile polyethylene bag (Tekmar Corporation, Cincinnati, OH), and homogenized with sterile saline for 30 s (Stomacher 80; Tekmar Corp.). Lung homogenate dilutions were prepared in sterile normal saline. Aliquots (100 μL) from homogenates and homogenate dilutions were plated on SBA and MacConkey agar plates and incubated at 35°C for 24 h. Carryover of the drug was controlled by serial dilution and by streaking of a small aliquot (100 μL) onto a large volume of agar (one full agar plate per 100 μL aliquot). The number of CFU of S. maltophilia was counted and recorded for each lobe, and the CFU per gram were calculated.

**(iii) Lung weights.** The total lung weight and pulmonary lesion scores are markers of organism-mediated tissue injury. The entire heart-lung block was carefully dissected and removed at necropsy, with attention being given to avoiding penetration of the tracheobronchial tree and pleural surfaces. The heart was dissected away from the lungs, leaving the tracheobronchial tree and lungs intact. The lungs were weighed (Mettler Instrument Co., Hightstown, NJ) and inspected by at least two blinded observers for the presence of lesions.

**(iv) Quantitative cultures of bronchoalveolar lavage fluid.** BAL fluid cultures were performed on each postmortem-excised tracheobronchial tree and lung preparation by the instillation of 10 mL of sterile normal saline into a clamped trachea with a sterile 10-mL syringe and subsequent withdrawal. The instillations were repeated twice for a total infusion of 20 mL. The lavage (8 to 14 mL) was then centrifuged for 10 min at 400 × *g*. BAL fluid supernatant was transferred into 2-mL Sarstedt microtubes, leaving 2 mL of supernatant and the pellet in the 15-mL Falcon tube, which was mixed by vortexing before performing quantitative cultures. For surviving rabbits, we performed a BAL fluid culture at the end of therapy (EOT), after euthanasia, and performed quantitative cultures from BAL fluid on SBA and MacConkey agar plates. For rabbits that did not survive until EOT, BAL fluid was obtained during the postmortem examination and also underwent quantitative cultures.

### Detection of emergence of resistance.

Organisms isolated from blood, BAL fluid, and lung tissue of rabbits in the cefiderocol treatment arms underwent antimicrobial susceptibility testing by BMD to assess for the emergence of resistance to cefiderocol. Similarly, organisms isolated from blood, BAL fluid, and pulmonary tissue of rabbits in the TMP-SMX treatment arm underwent antimicrobial susceptibility testing by BMD to assess for the emergence of resistance to TMP-SMX.

### Histopathological analysis.

Pulmonary lesions were excised and fixed in 10% neutral buffered formalin. Paraffin-embedded tissue sections were sectioned and stained with hematoxylin & eosin (H&E) and Brown and Brenn tissue Gram stain.

### Statistical analysis.

The following outcome variables among all experimental groups were compared: survival (days), lung weights (grams per tissue block), quantitative cultures of BAL fluid (log CFU per milliliter), quantitative cultures of lung tissue (log CFU per gram), and susceptibility of recovered organisms (assessment of emergence of resistance). Continuous variables were expressed as means ± standard error of the means (SEMs). The Kruskal-Wallis test was used to compare continuous variables among all groups, and Mann-Whitney U test was used for comparisons between two groups. Chi-square or Fisher’s exact test was used to compare categorical variables as appropriate. A two-tailed *P* value of ≤0.05 was considered to be statistically significant. Survival was plotted by Kaplan-Meier analysis and compared by the log-rank test.
